# Trafficking of Mononuclear Phagocytes in Healthy Arteries and Atherosclerosis

**DOI:** 10.3389/fimmu.2021.718432

**Published:** 2021-10-25

**Authors:** Lukas Tomas, Filip Prica, Christian Schulz

**Affiliations:** ^1^ Department of Medicine I, University Hospital, Ludwig Maximilian University, Munich, Germany; ^2^ DZHK (German Centre for Cardiovascular Research), Partner Site Munich Heart Alliance, Munich, Germany

**Keywords:** atherosclerosis, macrophage, monocyte, regression, trafficking

## Abstract

Monocytes and macrophages play essential roles in all stages of atherosclerosis – from early precursor lesions to advanced stages of the disease. Intima-resident macrophages are among the first cells to be confronted with the influx and retention of apolipoprotein B-containing lipoproteins at the onset of hypercholesterolemia and atherosclerosis development. In this review, we outline the trafficking of monocytes and macrophages in and out of the healthy aorta, as well as the adaptation of their migratory behaviour during hypercholesterolemia. Furthermore, we discuss the functional and ontogenetic composition of the aortic pool of mononuclear phagocytes and its link to the atherosclerotic disease process. The development of mouse models of atherosclerosis regression in recent years, has enabled scientists to investigate the behaviour of monocytes and macrophages during the resolution of atherosclerosis. Herein, we describe the dynamics of these mononuclear phagocytes upon cessation of hypercholesterolemia and how they contribute to the restoration of tissue homeostasis. The aim of this review is to provide an insight into the trafficking, fate and disease-relevant dynamics of monocytes and macrophages during atherosclerosis, and to highlight remaining questions. We focus on the results of rodent studies, as analysis of cellular fates requires experimental manipulations that cannot be performed in humans but point out findings that could be replicated in human tissues. Understanding of the biology of macrophages in atherosclerosis provides an important basis for the development of therapeutic strategies to limit lesion formation and promote plaque regression.

## Introduction

Atherosclerosis is characterised by a chronic, low-grade inflammation in the arterial wall. As the underlying pathology for myocardial infarction and stroke, it is the leading cause of death worldwide (1). The inflammatory response in the arterial wall is initiated by the hypercholesterolemia-induced subendothelial retention of apolipoprotein (apo)B-containing lipoproteins, mainly low-density lipoprotein (LDL), at sites of non-laminar and low shear stress blood flow. These sites are characterised by a higher abundance of macrophages (2–4), inflammation-primed endothelial cells (5) and particularly in humans a pro-retentive thickened intima rich in smooth muscle cells and altered extracellular matrix (6–8). The subendothelial retention makes the lipoproteins susceptible to enzymatic and non-enzymatic modification. In particular, oxidation of LDL triggers a sterile inflammatory reaction by activating the endothelial cells to upregulate adhesion molecules and secrete chemokines which attract monocytes and other leukocytes. Modification of lipoproteins also promotes their uptake by macrophages and vascular smooth muscle cells (VSMC) leading to the appearance of foam cells. Additionally, oxidized LDL contains several bioactive molecules, including oxidized phospholipids, which act as damage-associated molecular patterns (DAMPs), and together with early cholesterol crystal formation cause an activation of surrounding innate immune cells (9, 10). The continuous influx, retention and modification of apoB-containing lipoproteins, together with the defective resolution of inflammation and dysfunctional clearance of dead cells (efferocytosis) fuel the chronic inflammation (11). The persistent inflammatory activity also leads to the generation of autoantigens and involvement of the adaptive immune system at later stages of the disease (12, 13).

Resident arterial macrophages play a crucial role in tissue homeostasis and serve as immune sentinels within the tissue. Adventitial macrophages, for instance, are important regulators of collagen production and the arterial tone (14). At areas of low blood velocity and shear stress, macrophages beneath the endothelium survey the environment to detect pathogens or potentially hazardous deposits (15). As such, aortic intima-resident macrophages are among the first cells to encounter trapped apoB-containing lipoproteins at the initiation of hypercholesterolemia (2–4). Mainly based on their expression of CD11c, these subendothelial phagocytes were initially described as dendritic cells, but recent results have challenged this view and have identified macrophages as the main cell type to first encounter the trapped lipids (16). Furthermore, in mice with a deficiency of monocytes and macrophages, a delayed and almost abolished development of atherosclerotic plaques can be seen (17–21). This further underlines the importance of the monocyte-macrophage axis in the initiation of atherosclerotic disease. With the development of mouse models for atherosclerotic regression, it has become clear that macrophages are not only important drivers of the disease, but their plasticity and diverse repertoire of homeostatic functions also makes them important effectors in atherosclerotic regression (22).

Since the description of the Mononuclear Phagocyte System, the prevailing paradigm was that tissue resident macrophages are continuously seeded from circulating monocytes. In recent years, however, it has become obvious that under homeostasis the tissue macrophage pool is mainly maintained through local proliferation and does not solely depend on monocyte influx (23–25). Monocyte-independent seeding of resident tissue macrophages starts early in embryonic development. Macrophages originating from the extra-embryonic yolk sac (YS) populate tissues during embryonal development as erythro-myeloid progenitor (EMP)-derived macrophages and persist into adulthood (26, 27). Microglia in the central nervous system are for instance exclusively derived from YS progenitors, without input from blood monocytes (28, 29). However, in most organs, a second wave of monocyte-derived macrophages, originating from definitive haematopoietic stem cells within the fetal liver and bone marrow (BM), co-colonize the tissues (30, 31). The question of tissue macrophage ontogeny has critical implications. EMP-derived macrophages migrate to tissues at the time of organogenesis and seem indispensable in various developmental processes (32–36). This developmental and homeostatic function might prevail in adult life, generating an important link between macrophage ontogeny and function. Indeed, we and others have found that macrophages of different ontogeny perform distinct tissue-specific functions and maintain a specific phenotype (37–41). Delineating monocyte-macrophage ontogeny and trafficking might improve our understanding of the maladaptive chronic inflammatory response in atherosclerosis development, as well as their role in atherosclerosis regression. Ultimately, this could lead to targeted approaches tackling the high rates of global cardiovascular mortality and morbidity resulting from atherosclerosis.

In this Review we address the knowns and unknowns of the trafficking, dynamics and fates of vascular monocytes and macrophages in the healthy and atherosclerotic aorta. Analysing these properties in human tissues is complicated by the availability of human material and models. Therefore, we will focus primarily on results from the mouse as a model organism but put these results into human context where possible at the end of this Review.

## Monocytes and macrophages in the healthy aorta

The development and broad accessibility of novel high-dimensional analysis techniques, such as multi-parameter flow cytometry, single-cell RNA sequencing (scRNA-seq) and cytometry by time of flight, has enabled scientists to obtain a clearer picture of leukocyte diversity in the healthy mouse aorta. These studies revealed that myeloid cells, and in particular macrophages, are the dominant immune cell type in the healthy arterial wall (16, 37, 42, 43). Arterial macrophages are primarily located in the fibrous outer arterial layer, the adventitia. Only a small number of macrophages can be found in the innermost layer, the intima, just below the endothelial cells. Based on histological and scRNA-seq data, it’s estimated that up to 10% of the arterial macrophages are located in the intima, whereas 90% are positioned within the adventitial layer (16, 37, 44).

**Figure 1:**
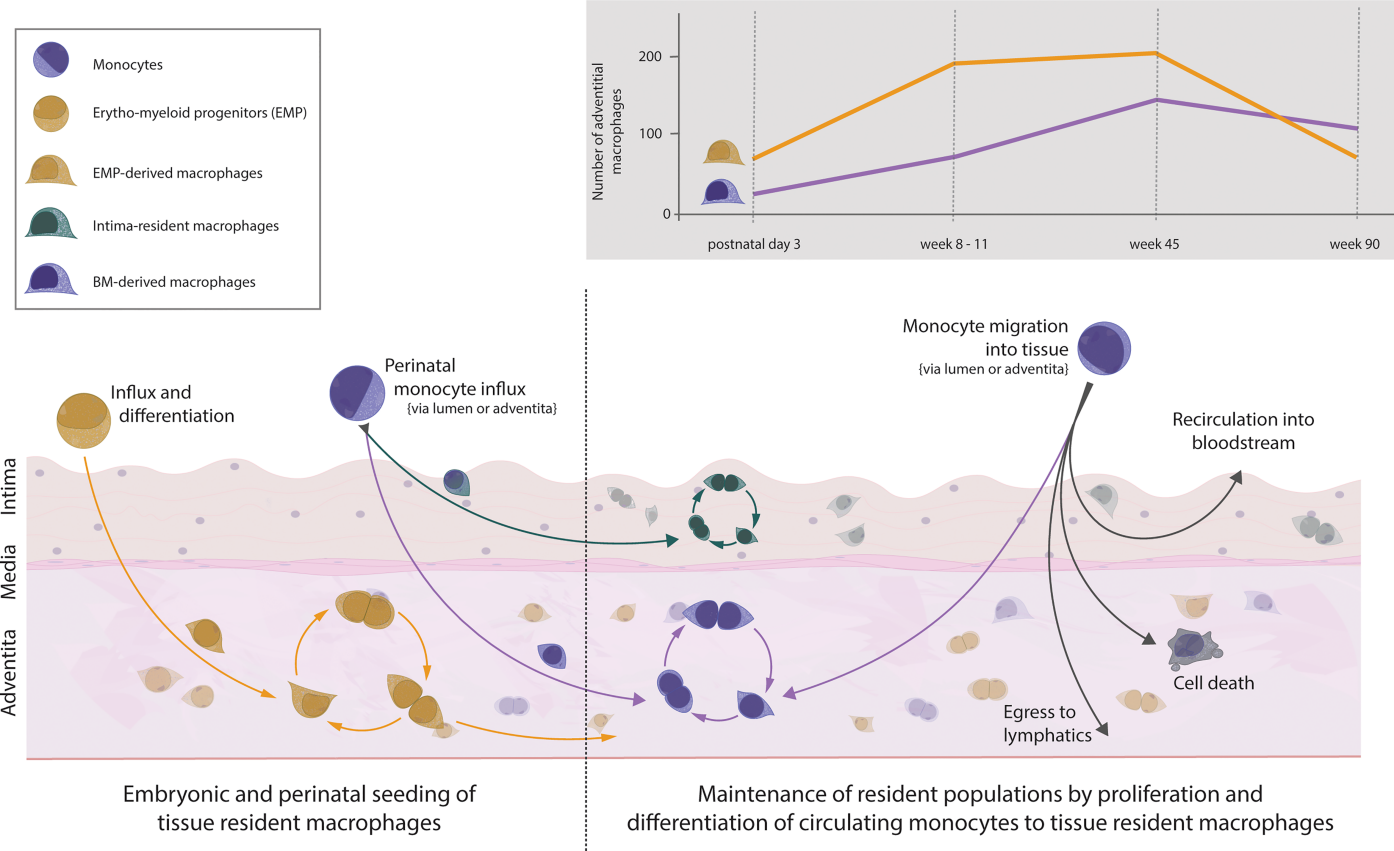
Vascular macrophages and monocytes in the healthy mouse aorta. Influx of EMP-derived macrophages into the tissue during embryogenesis starts around embryonic day 9.5. Macrophages settle within the aortic adventitia and sustain solely through local proliferation. Around embryonic day 18.5, monocytes from the bone marrow seed the aorta and differentiate within the adventitia (forming a population of BM-derived tissue resident macrophages) as well as within the intima (forming a separate population of intima-resident macrophages). This recently defined population of intimal macrophages is heavily seeded perinatally but maintains solely through local proliferation. Thus, the adventitia is colonised with macrophages of dual origin which self-sustain numbers through proliferation, and in the case of adventitial BM-derived cells, replenishment from circulating monocytes. The number of cells of different ontogeny varies throughout life, with numbers changing in age adopted from (37). The fate of monocytes migrating into the steady-state aorta follows several possible fates: differentiation into BM-derived macrophages, further migration towards lymphatics, apoptosis, or migration back into circulation.

The aorta is populated with macrophages early on during embryonic development. Macrophages can be observed in the fetal aorta at embryonic day 16.5 and most likely start inhabiting the niche from embryonic day 9.5 onwards (27, 32, 44). This prenatal wave of macrophages colonising the aorta is dominated by YS EMP-derived macrophages which travel to the aorta without a monocyte intermediate. After birth, the brief influx of blood monocytes, which consequently differentiate into tissue resident macrophages, contributes to the aortic macrophage pool (37, 44). Despite the monocytic influx, YS EMP-derived macrophages are not replaced by BM-derived macrophages, as has been suggested previously. Rather, the entire adventitial macrophage pool of EMP- and BM-derived macrophages continues to expand in numbers until 45 weeks of age, with YS EMP-derived macrophages being the dominant tissue-resident macrophage population (**Figure 1**). In aged mice, at around 90 weeks, a general drop of adventitial macrophage numbers mainly affecting EMP-derived macrophages can be observed (37). In contrast to adventitial macrophages, macrophages residing in the intima have recently been reported to seed almost exclusively after birth (16). Using various mouse models, including CD115, CX3CR1 and Flt3 reporter mice, Williams et al. showed that the macrophages inhabiting the intimal layer originate exclusively from definitive haematopoiesis. Interestingly, intimal macrophages are primarily found in locations of increased hemodynamic stress, which are predilection sites for atherosclerosis development (2, 3). Although we did not specifically investigate this aspect, our results show no site-specific tropism of adventitial macrophages throughout the aortic segments, in contrast to intimal macrophages (37). This puts further emphasis on understanding the origin, dynamics, and function of intima-resident macrophages. Given the critical role of intimal macrophages in atherosclerosis development, it will be interesting to see the results by Williams et al. confirmed with more efficient fate-mapping models, such as the recently generated Rank^Cre^ (45) and Ms4a3^Cre^ mice (30), in a quantitative approach.

Of note, one group has identified an unconventional Sca-1^+^CD45^+^ cellular subset in the adult murine aorta, proposed to be adventitial macrophage-committed progenitor cells (46, 47). A recent scRNA-seq study also identified an interferon-poised subset of Sca-1^+^ aortic macrophages, whereas the macrophage progenitor potential of aortic cells has been questioned by others (48, 49). Whether these Sca-1^+^CD45^+^ cells are indeed a macrophage progenitor population or potentially are provided by Sca-1^+^ mesenchymal stem cells remains to be elucidated (43, 50–54).

Under homeostasis, the adult arterial macrophage pool experiences little dynamic. Adventitial EMP- and BM-derived macrophages self-maintain with minimal input from monocytes entering the arterial wall. Using irradiation-free chimeras and parabionts, we and others have found that over a period of 9 months, only 20% of the arterial macrophages are replenished by monocytes (37, 44). In addition, this number seems to be a constant, as we observed a similar 20% monocyte input after a 3-month observation period (37). Contrary to the macrophages residing in the adventitia, the intimal macrophages appear to not be replaced by monocytes under homeostasis (16).

Besides the quantitatively limited replenishment of the macrophage pool in the adventitia, monocytes have important homeostatic functions in the vasculature. Non-classical Ly6C^low^CCR2^-^ monocytes, which derive from Ly6C^high^CCR2^+^ in mice, crawl along the endothelium to survey the cellular integrity and sense dangers, as well as to remove cellular debris (55–57). Ly6C^low^ monocytes are, however, thought to rarely cross the endothelial barrier into the tissue (57, 58). In contrast, classical Ly6C^high^ monocytes are highly mobile and extravasate, mainly guided by their CCR2 expression. A population of transiently sessile monocytes has been found in the lungs and skin of mice in their steady-state (59). These ‘tissue monocytes’ have previously also been identified in the spleen, which acts as a reservoir to quickly mobilize immune cells upon inflammation (60). Contrary to splenic monocytes, the Ly6C^high^ monocytes in lung and skin can survey the tissue environment and transport antigens to lymph nodes (59). Although the question of sessile monocyte existence has not been addressed for the arterial wall, monocytes are readily identified in the healthy arterial wall (3, 42, 43, 61). Their homeostatic turnover and ability to recirculate into the blood or leave *via* afferent lymphatics into adjacent lymph nodes, similarly to the surveying monocytes in lung and skin, remains to be determined.

The high motility of Ly6C^high^ monocytes comes with the price of potentially spreading infectious agents (62, 63). This might in part explain the presence of infectious agents within atherosclerotic plaques (64). A remaining question is the location of vessel wall entry of Ly6C^high^ monocytes. It has not yet been clarified if and to what extent classical Ly6C^high^ monocytes enter the healthy vascular wall *via* vasa vasorum in the adventitia or from the luminal side. This is of particular interest in advanced plaques, where intra-plaque sprouting of leaky vessels occurs and could drive the chronic inflammation through the constant supply of monocytes (65).

Analogous to the heterogenous homeostatic functions of monocytes, we and others have found that resident adventitial macrophages have a diverse functional outfit. Traditionally, macrophages have been divided in classically activated M1 and alternatively activated M2 macrophages (66). These two states are, however, *in vitro*-based extremes on opposite poles of a continuum of macrophage functionality. Novel multiparametric analysis methods have established the high plasticity and different nuances in the macrophage functional outfit (67–69), also in the aortic wall. Recent integrated analyses of scRNA-seq datasets from healthy and atherosclerotic mouse aortas revealed the presence of 5 major macrophage subsets (70, 71). As described in more detail below, these subsets are (I) inflammatory, (II) triggering receptor expressed on myeloid cells 2 (TREM2)^+^, (III) interferon inducible, (IV) resident-like and (V) cavity macrophages. These five subsets can be found both in the atherosclerotic and healthy aorta, although the complexity of macrophage phenotypes increases in atherosclerotic aortas (70, 71). Strikingly, by employing scRNA-seq in Rank^Cre^Rosa26^eYFP^ mice we were able to map the transcriptional heterogeneity in adventitial macrophages to their origin. The healthy mouse adventitia harbours a macrophage subset with a homeostatic and anti-inflammatory transcriptional profile that derives almost exclusively from YS EMPs. These macrophages were characterised by a high expression of the hyaluronan receptor encoding gene Lyve-1, a known marker for resident macrophages, which maps them to the macrophage subset responsible for the regulation of aortic collagen production (14), and to the resident-like macrophages described above. Furthermore, EMP-derived macrophages expressed high levels of stabilin 1 (Stab1) and growth arrest specific 6 (Gas6), both of which are important for efferocytosis, a process crucial for the inhibition of atherosclerosis (37, 72–74). In contrast, a cluster that lacked *eYFP* transcript expression and was comprised of monocyte-derived macrophages expressed gene sets that were associated with pro-inflammatory properties, including *Il1β* (37), similar to the subset of inflammatory macrophages. Thus, there seems to be a division of labour in arterial macrophage subsets of different ontogeny, where EMP-derived macrophages are responsible for homeostatic processes like collagen production and efferocytosis. BM-derived macrophages in turn are in a poised state for defending the arterial integrity against pathogens. Thus, it would not be surprising if macrophages of diverse origins play different roles during atherosclerosis progression and regression, given their distinct set of functions.

## Enhanced Monocyte Influx and Macrophage Proliferation During Atherosclerosis Development and Progression

The intima-resident macrophages are among the first cells exposed to the increased influx of apoB-containing lipoproteins during hypercholesterolemia. These cells are critical in atherosclerosis initiation. The aorta of mice engineered to lack aortic intima-resident macrophages displays decreased lipid deposition in the early stages of atherosclerosis (4, 16). Within days of sustained hypercholesterolemia, the capacity of macrophages to metabolize the accumulating lipids and cholesterol is overwhelmed. This leads to the deposition of lipid droplets within the macrophage cytoplasm, resulting in the typical foam cell appearance, and even macrophage death. Macrophage death and defective clearance are known to be major drivers of the atherosclerotic process (16, 75, 76).

Initially, foam cells appear to be exclusively derived from resident intimal macrophages in the mouse (16). Of note, in humans, VSMCs also play a role in the early development of foam cells (77). Continuous inflammatory triggering by the persistent influx of apoB-containing lipoproteins causes a substantial monocyte recruitment within the first 1-2 weeks of hypercholesterolemia (16, 78, 79). The subendothelial inflammatory foci lead to the expression of adhesion molecules on activated endothelial cells and the secretion of chemokines, most importantly of CCL2/MCP-1, CX3CL1 and CCL5 (80) These factors are essential for the infiltration of primarily Ly6C^high^ monocytes into the developing atherosclerotic plaque (81, 82). Combined absence of all three chemokine-chemokine receptor pairs results in an almost complete inhibition of lesion development (82–84). Intravital imaging studies suggest that the luminal (‘inside-out’) recruitment is important in the early phases of plaque development, whereas (‘outside-in’) recruitment *via* adventitial vasa vasorum is the main route for myeloid cells to enter advanced plaques (78, 85). More quantitative approaches with adoptive transfer of monocytes and bead labelling found that both the influx and luminal recruitment routes are important already in early atherosclerotic development, and persist in advanced plaques (86, 87). The route of plaque-invading monocytes is an important avenue of research, as these cells have been recognized to fuel the inflammatory reaction in developing plaques and blocking their entry might represent a promising therapeutic target.

In addition to causing a local inflammatory responses and recruitment of Ly6C^high^ monocytes into the vessel wall, hypercholesterolemia induces a Ly6C^high^ dominated monocytosis (81, 82). Elevated levels of cholesterol in haematopoietic stem cells foster the formation of lipid rafts and stabilisation of growth factor receptors, which promote their myelopoietic activity and monocytosis (88–90). Supplementing the enhanced myelopoiesis in the bone marrow, extramedullary haematopoiesis in the spleen contributes to increased production of monocytes and marked recruitment into the developing atherosclerotic lesion (60, 91). Other lifestyle-related factors such as hyperglycaemia or stress also have the potential to enhance myelopoiesis and fuel the cycle of monocyte production and entry into the plaque (92–94). Importantly, the circulating monocytes are poised for pro-inflammatory reactions with increased levels of surface receptors such as CD86 and TLR4, as well as increased levels of reactive oxygen species, among other features (44, 95–97). Thus, hypercholesterolemia leads to augmented recruitment and an increased number of circulating monocytes with a heightened inflammatory potential. A topic that warrants further investigation is the role of recently identified monocyte subsets that appear during inflammatory conditions, such as segregated-nucleus-containing atypical monocytes (98), in atherosclerosis development.

**Figure 2:**
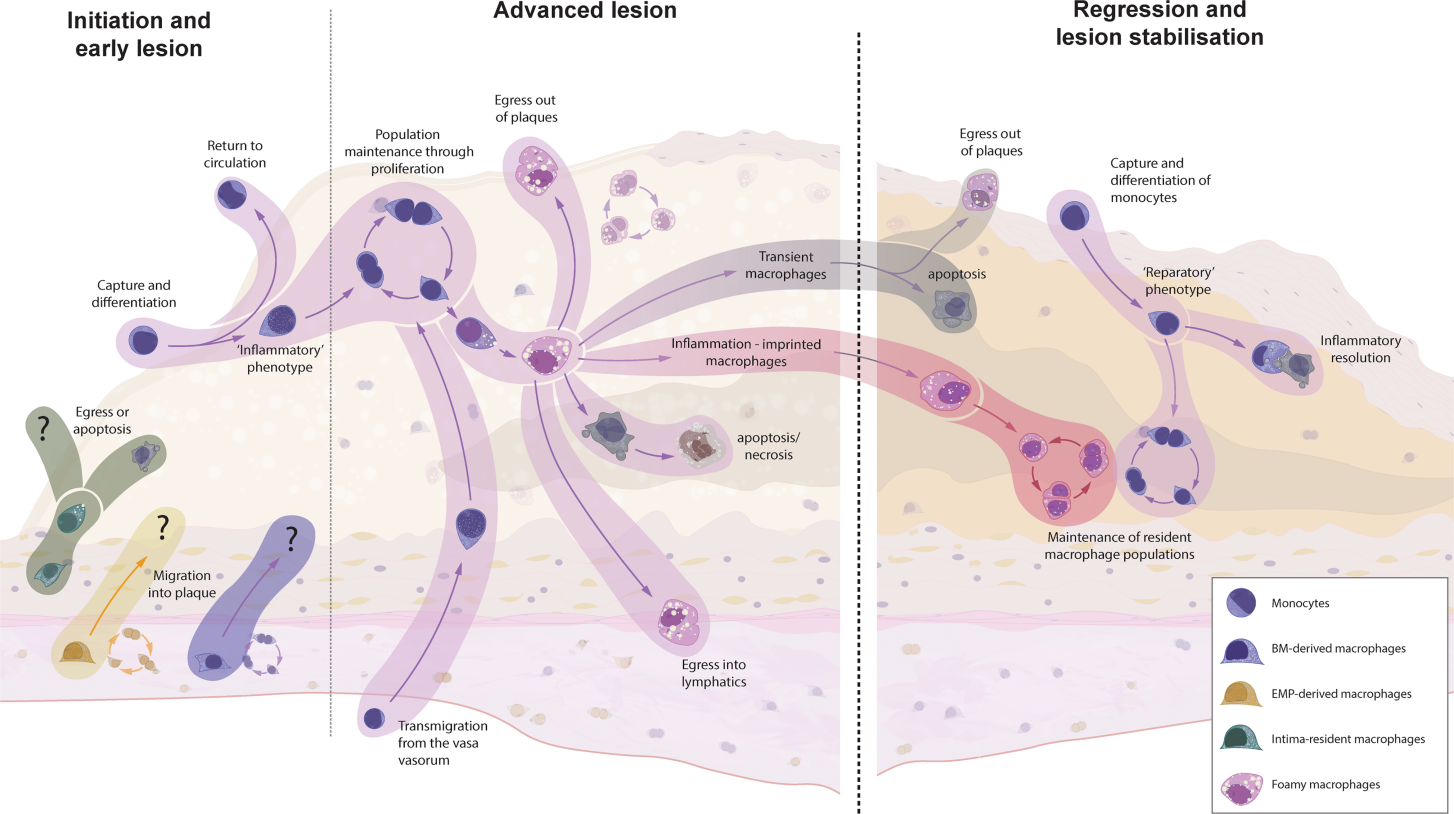
The origin and fate of macrophages in murine models of atherosclerotic plaque formation and regression. Intima-resident macrophages are the first to encounter accumulating apoB-containing lipoproteins but are replaced by recruited macrophages within weeks. It is unknown whether resident adventitial (EMP- and BM-derived) macrophages can invade the intima at any point of atherosclerosis progression or regression. Monocyte recruitment is the dominant source for plaque macrophages during early atherosclerosis, whereas local macrophage proliferation takes over at later stages. In other inflammatory disorders, macrophages can migrate either transendothelially or *via* lymphatics to clear the inflammatory triggers and present them to the adaptive immune system. However, hypercholesterolemia supresses emigration, leading to a continuous accumulation of cells, resulting in increased apoptosis and secondary necrosis. Upon the cessation of hypercholesterolemia, the fate of localised macrophages is yet unknown. Research from other inflammatory disorders has shown that, upon removal of the inflammatory stimuli, a number of macrophages will clear through apoptosis (transient macrophages), but inflammatory imprinting can define a population of surviving macrophages which have an acquired epigenetic memory - which might have detrimental effect on disease resolution and recurrence of inflammation. Additionally, a novel wave of monocyte recruitment defines a fresh population of reparatory macrophages, which aid in tissue clearance and tissue repair.

The recruited Ly6C^high^ monocytes are thought to primarily differentiate into intimal macrophages (75). Data from developing atherosclerotic plaques is lacking, but it is conceivable that Ly6C^high^ monocytes have alternative fates within the lesion (**Figure 2**). As has been shown for sterile liver injury, Ly6C^high^ monocytes can exhibit distinct monocyte-specific functions, including the uptake of trapped apoB-containing lipoproteins (99–101). In this way, monocytes participate in the vicious cycle of cellular apoptosis and necrosis following the metabolic stress of intracellular cholesterol accumulation. Some Ly6C^high^ monocytes might also recirculate into the blood and lymph and present antigens, including *de novo* generated autoantigens to the cells of the adaptive immune system (12, 13, 59). Ly6C^low^ monocytes, on the other hand, show an intensified patrolling behaviour at atheroprone sites, which display increased endothelial damage during hypercholesterolemia (58, 102). Despite their main task as patrolling intravascular cells, Ly6C^low^ monocytes can also be found in the atherosclerotic plaque, highlighting their potential to extravasate – although to a lesser extent than classical Ly6C^high^ monocytes (70, 82). These cells display an anti-inflammatory transcriptional signature with elevated transcripts for cholesterol efflux and vascular repair, thereby promoting the inflammation resolution (58). It is still debated whether the extravasation of Ly6C^low^ monocytes is of importance in the atherosclerotic disease process (58). If so, the anti-atherosclerotic phenotype of Ly6C^low^ monocytes presumably ameliorates the disease process and enhancing Ly6C^low^ monocyte extravasation might be a potential therapeutic target.

Akin to other chronic inflammatory diseases, the initial, mainly CCR2-dependent, recruitment of monocyte-derived macrophages is a crucial pathomechanism in the development of atherosclerosis (82–84, 103–106). However, as the atherosclerotic lesion progresses, monocyte recruitment becomes less important, as evidenced by studies with CCR2- or monocyte-depletion models (21, 107–109). The limited impact of blocking monocyte recruitment on progression of advanced plaques might be due to a failure of monocytes to penetrate the lesion. A recent report suggests that monocytes cannot migrate deeply into the lesion and only accumulate superficially, similar to tree ring formation (87). This is, however, contradicted by results showing migrating CD11c^+^, which appear to be similar to foamy monocytes or macrophages, within atherosclerotic plaques (100, 101, 110). Consequently, there might be other reasons for the non-reliance on monocyte entry in progressing plaques, such as local macrophage proliferation, as discussed below.

Hypercholesterolemia leads to a substantial influx of monocytes. It has recently been suggested that the perinatally seeded intima-resident macrophages are completely replaced by invading monocyte-derived macrophages within weeks of hypercholesterolemia (16). Although similar results have been observed for liver Kupffer cells during Listeria infection (111), this contrasts with the fate of adventitial macrophages in other models of sterile and non-sterile aortic inflammation. We and others found that, after a transient recruitment of monocyte-derived macrophages in the acute phase, the resident macrophage population prevails even in chronic inflammatory models (37, 44). Our results focused on adventitial macrophages, but the different response of intimal macrophages in atherosclerosis is an intriguing characteristic that might be contributing to the defective inflammation resolution in atherosclerosis (61, 81). Given that in mice the entire adventitial macrophage pool requires approximately one year for a complete cell turnover (44), it is likely that the turnover of intimal macrophages during hypercholesterolemia is accelerated by mechanisms like emigration or cell death, which in turn could fuel atherosclerotic development. Nonetheless, it is possible that intima-resident macrophage numbers also rebound after the cessation of hypercholesterolemia, but this remains to be elucidated.

The fate of the intima-resident macrophages is of particular interest in the context of atherosclerosis, as we know from other inflammatory conditions that most monocytes recruited under inflammatory conditions do not stably engraft as resident macrophages. These transient macrophages disappear upon the resolution of inflammation (25, 39, 112, 113), which has also been shown in the aorta (44). Future studies will have to elucidate the macrophage composition and origin within the intimal niche after the cessation of hypercholesterolemia. Studies focussing on the lung and other tissues have also found that some *de novo* recruited macrophages are not transiently resident but persist even after inflammation resolution. Importantly, these macrophages were shown to acquire an epigenetic memory of the inflammatory situation (inflammation-imprinted resident macrophages), which might have detrimental effects on tissue repair or repetitive insults (113). Whether the newly recruited atherosclerotic macrophages share fates with inflammatory macrophages in other tissues and vanish after removal of the inflammatory stimulus is the topic of ongoing research.

Despite the increased macrophage apoptosis and necrosis in atherosclerotic plaques, macrophage numbers are stable throughout disease progression (49). It has been estimated that in early atherosclerosis monocyte recruitment accounts for approximately 70% of the macrophage replenishment, while more than 85% of the macrophages in advanced plaques stem from *in situ* proliferation (49). Interestingly, whereas proliferation of intimal macrophages increases as the atherosclerotic lesions progresses, adventitial macrophages do not proliferate more, as if they were not affected by the ongoing inflammatory process (49). Macrophage loss in atherosclerotic plaques is mainly a result of cell death. In infectious settings, macrophages emigrate from the site of infection either *via* reverse transendothelial migration or *via* lymphatics to clear the inflammatory triggers and present them to the adaptive immune system (62, 114). Hypercholesterolemia, however, suppresses emigration signals *via* CCR7 and macrophage migratory capacity, leading to a continuous accumulation of macrophages and increased local cell death with the development of a necrotic core (62, 115–118). In general, the migration behaviour of plaque macrophages has been characterised as ‘dancing on the spot’, i.e. macrophages do not migrate within the plaque but only extend and retract their dendrites (87, 110, 119). This inability to migrate begs the question whether resident adventitial macrophages are capable of crossing the muscular media and migrate into the developing plaque.

Phenotype and functions of macrophages are governed by transcriptional regulation. It has been suggested that the transcriptional programs of intima-resident macrophages and recruited monocyte-derived macrophages converge on a similar foamy macrophage profile early in hypercholesterolemia (16, 71). But developing atherosclerotic plaques harbour many heterogenous subsets of macrophages. As described above, efforts to integrate the various scRNA-seq studies of the murine atherosclerotic plaque have defined 5 distinct macrophage subsets: (I) inflammatory, (II) TREM2^+^, (III) interferon inducible, (IV) resident-like and (V) cavity macrophages (70, 71). Inflammatory macrophages show elevated expression levels of pro-inflammatory cytokines like interleukin 1β and tumor necrosis factor. The strong pro-inflammatory gene profile and the importance of interleukin 1β in atherosclerotic disease points towards a major role of this macrophage subset in aggravating the chronic atherogenic inflammation. These cells were furthermore characterized by high CCR2 expression and presumably are transient inflammatory macrophage descendants from invading Ly6C^high^ monocytes. Macrophages expressing TREM2 have been identified as the foam cell population in atherosclerotic plaques (120–122). TREM2 is a transmembrane glycoprotein that can interact with apolipoprotein E and TREM2^+^ macrophages show a transcriptomic signature enriched for lipid metabolism pathways, pinpointing their role in lipid and cholesterol handling (38, 123). TREM2^+^ macrophages have previously been shown to possess anti-inflammatory functions (124). The TREM2^+^ macrophage subset in atherosclerotic plaques is also characterized by dramatically decreased expression levels of pro-inflammatory molecules like interleukin 1β, tumor necrosis factor or NLR family pyrin containing domain 3 (Nlrp3) (120, 121). Furthermore, TREM2^+^ macrophages have been found to express increased levels of CD11c (110), similar to foamy monocytes (100, 101). Interestingly, TREM2 expression is found in a variety of disease-associated macrophages, including microglia during neurodegenerative disease and lipid-associated macrophages in obesity (38, 125). Even in our dataset of adventitial macrophages during angiotensin II-induced arterial inflammation, we were able to identify TREM2^+^ macrophages (71). Consequently, TREM2^+^ macrophages might represent a phenotype that is associated with tissues exhibiting increased lipid deposition and apoptosis. In both scenarios, macrophages capable of handling lipid depositions are required for tissue homeostasis. Additionally, apoptotic cell death, which leads to increased lipid and cholesterol deposition, is associated with anti-inflammatory cell functions (76). It is interesting to note that TREM2^+^ macrophages do not seem to be related to only one ontogeny but can derive from both YS and BM (38, 71, 125). The interferon-inducible macrophages are a rather small subset in the atherosclerotic plaque (70). These macrophages are characterised by expression of several interferon-inducible genes, including *Isg15* and *Irf7*. Future studies will have to investigate this so far unknown subset but given the pro-atherosclerotic role of type I interferon signalling, interferon-inducible macrophages might be detrimental in the course of the disease (126). The identified resident-like macrophage subset is characterised by high expression of *Lyve-1*, a gene that is important in resident adventitial macrophages for regulation of collagen production in the arterial wall (14). Similar to our dataset of Lyve-1 expressing macrophages in the healthy aorta (37), resident-like macrophages in the atherosclerotic plaque showed increased gene expression of Gas6 (48, 70, 121). Consequently, resident-like macrophages might be important in the efferocytotic clearance of apoptotic plaque cells and thus be major influencer of the balance between progressing and regressing atherosclerotic plaques. A drawback of the scRNA-seq studies is that we cannot distinguish between intimal and adventitial macrophages. Thus, the presence of resident-like macrophages in atherosclerotic aortas does not provide evidence for a role of Lyve-1^+^ resident macrophage in the intima-focussed atherosclerotic disease process. The discovery of a macrophage subset expressing a gene signature reminiscent of ‘cavity macrophages’ is an interesting aspect, in light of recent reports. Mature macrophages from serous cavities like the peritoneum or pericardium have been shown to invade surrounding tissue during sterile inflammation where they play important roles during tissue repair (127–129). The presence of cavity macrophages in atherosclerotic aortas indicates that adjacent macrophages, from serous cavities or potentially even the adventitia, can invade the atherosclerotic aorta and intima.

Even though resident adventitial macrophages constitute 90% of the aortic macrophages and are the only arterial subset originating from EMPs, their involvement in the atherosclerotic disease process is unclear. The role of adventitial macrophage subsets, including the YS EMP-derived adventitial macrophages, warrants further investigation. As described above, EMP-derived adventitial macrophages show a distinct transcriptional signature of anti-inflammatory and efferocytic functions, that is preserved during chronic arterial inflammation (37). Failing efferocytosis, in particular, has been shown to be a major pathogenic factor in atherosclerotic development (76). Adventitial EMP-derived macrophages seem to be predestined to counteract this failure and inhibit the inflammatory cycle within atherosclerotic plaques – if they invade the growing lesion. As outlined herein, plaque macrophages show diminished migratory behaviour, and adventitial macrophages are not thought to invade the growing plaques. On the other hand, the presence of cavity macrophages suggests that certain macrophage subsets might still be able to invade the developing lesions. Also, a CD11c^+^ cell subset, which resemble foamy macrophages, has been shown to be actively migrating within the plaque. These results warrant further investigation on the trafficking of adventitial macrophages and macrophages of different ontogenies during the various stages of atherosclerosis development.

## Macrophage Egress and Monocyte Migration in Regressing Atherosclerotic Plaques

Atherosclerosis is characterised by a failure to resolve the inflammatory response. The continuous influx and retention of apoB-containing lipoproteins represents a persistent inflammatory stimulus. The lowering of blood lipid levels, in particular cholesterol levels (130), allows the resolution phase to commence. The resolution or regression of atherosclerotic plaques can lead to a reduction of plaque size, but most importantly results in the scarring and stabilisation of advanced lesions, lowering the risk of myocardial infarction and stroke (22). The reduction of plaque leukocyte abundance and a phenotypic switch in plaque cells are important hallmarks during atherosclerosis regression (131). Macrophages are highly plastic cells, and as such are fundamental players in the tissue repair processes seen in atherosclerosis regression.

Traditional mouse models of atherosclerosis, like the LDL receptor and apolipoprotein E knockout mouse, have greatly contributed to our understanding of the atherosclerotic disease process. These models, however, lack the ability to normalise hypercholesterolemia and induce regression. Fortunately, in recent years several mouse models of atherosclerosis regression were developed (132–138). The common denominator of these models is the normalisation of cholesterol levels after a phase of hypercholesterolemia to induce advanced atherosclerotic plaques. Examples include the transplantation of atherosclerotic aortic segments into normocholesterolemic mice or the inducible deficiency of the microsomal triglyceride transfer protein, as in the Reversa mouse (132, 133). The variety of regression models, as well as their individual limitations, such as surgical inflammation and a lack of lymphatic anastomosis in the transplantation model might be the reason for the heterogeneous results regarding the fate of macrophages in atherosclerosis regression. A novel approach uses antisense oligonucleotides, targeting the LDL receptor to transiently cause hypercholesterolemia and induce atherosclerotic plaques. In this model, regression can be induced either by discontinuing the antisense oligonucleotides or through treatment with sense oligonucleotides for the LDL receptor (136). The LDL receptor antisense method offers a promising approach, as it allows scientists to omit time- and labour-intensive cross-breedings when using transgenic animals in regression. Furthermore, due to its limited off-target effects, antisense treatment is even used in human hyperlipidaemic disease (139, 140).

A hallmark of atherosclerosis regression is the reduction of the plaque macrophage content (87, 117, 141–146). Macrophage emigration from arteries *via* afferent lymphatics or reverse transendothelial migration aids the host defence by presenting antigens to the adaptive immune system (62, 114). As described above, hypercholesterolemia blunts the CCR7-guided emigration *via* the expression of neuroimmune guidance cues, including netrin 1 and semaphoring 3E, and by increasing plasma membrane cholesterol content which affects intracellular signalling as well as other mechanisms (62, 115–118, 147). Not surprisingly, the reversal of hypercholesterolemia has been shown to induce CCR7 expression in plaque macrophages and with it their efflux *via* afferent lymphatics (117, 142–144, 148–150). Whether lesional macrophages leave the regressing plaque *via* reverse transendothelial migration, as well as the quantitative relevance of macrophage emigration to the overall loss of plaque macrophages has not yet been clarified. Increased macrophage emigration has been observed in several different models of atherosclerosis regression, including the aortic transplantation, the Reversa mouse and apoB-targeted antisense oligonucleotide treatment (117, 142–144, 148–150), whereas other reports have found no difference in macrophage emigration behaviour during regression (87, 145, 146). Importantly, emigration of plaque macrophages to lymph nodes might aid the development of the recently described post-resolution phase, although it is unknown whether this establishment of adaptive immunity takes place in atherosclerosis regression (151).

Another common mechanism of leukocyte removal during tissue repair is programmed cell death *via* apoptosis (152). Effective clearance of apoptotic cells by macrophages avoids secondary necrosis and suppresses inflammation. Additionally, efferocytosis aids tissue repair by inducing a pro-resolving phenotype in phagocytosing macrophages (76). Whereas a recent report identified increased macrophage apoptosis as part of the regression mechanism (149), other studies did not find elevated numbers of apoptotic macrophages in regressing plaques (145, 146, 150). In order for apoptosis to act as a pro-resolving stimulus, efferocytosis needs to be functional. In atherosclerosis progression, however, defective efferocytosis is an essential pathogenic mechanism (11). The role of macrophage apoptosis in atherosclerosis regression remains elusive, and further studies investigating the presence and functionality of efferocytosis in atherosclerosis regression are warranted.

As macrophage numbers in advanced atherosclerotic plaques are primarily maintained through local proliferation, another means of reducing the plaque macrophage burden is through the suspension of proliferation. Indeed, a decrease in proliferating macrophages can be observed within 3 weeks of regression (145, 149). An inhibition of macrophage proliferation upon cessation of hypercholesterolemia is not an unexpected finding, as the retained and modified apoB-containing lipoproteins are potent inducers of M-CSF, contributing to an increase in local macrophage proliferation in advanced plaques (49, 153, 154).

In addition to macrophage survival and proliferation, monocyte recruitment is another factor influencing plaque macrophage numbers. The reversal of hypercholesterolemia presumably blunts the heightened monocytopoiesis and normalises circulating Ly6C^high^ monocyte levels. However, so far, no difference could be detected in studies evaluating the monocyte frequency even after 4 weeks of regression (145, 146). These intriguing results warrant further studies focusing on the timing and return to a steady-state haematopoiesis following the onset of normocholesterolemia. Nonetheless, monocyte extravasation is not only dependent on the number of circulating monocytes, but also on their potential to invade the regressing plaque. Similarly to mechanisms halting proliferation of plaque macrophages, decreased *de novo* generation of macrophages from immigrating monocytes would result in a reduction of plaque macrophage abundance. Experimentally, several groups have detected a suppressed migration of Ly6C^high^ as well as Ly6C^low^ monocytes into the regressing plaque by using the adoptive transfer of labelled monocytes, as well as by monocyte tracking with fluorescent beads (145, 146, 155). The quantitative relevance of this effect might, however, be limited. Härdtner et al. estimated that the limited monocyte recruitment accounts for only about 25% of plaque macrophage reduction (145), whereas another report found no suppression of monocyte influx in regressing plaques, despite using similar methods (149). In summary, there are various mechanisms at play reducing the abundance of inflammatory macrophages in regressing atherosclerotic plaques. Presumably, all four mechanisms mentioned herein are relevant for ameliorating the inflammatory burden, likely occurring at various stages of regression. Longitudinal studies of macrophage trafficking, in combination with fate-mapping models and other methodologies capable of tracing the fates of lesional macrophages will hopefully advance our understanding of the cellular dynamics in regression.

The diminished monocyte influx during atherosclerosis regression is an interesting avenue for further research. The resolution and repair phase after myocardial infarction, as well as following sterile injuries in other organs, depends on the continuous influx of monocytes, which consequently differentiate into reparatory and pro-resolving macrophages (156–160). The importance of monocyte migration into the arterial wall to facilitate inflammation resolution and tissue repair has recently also been established for atherosclerosis regression. Applying the aortic transplantation models in numerous chemokine receptor knockout and reporter mice, Rahman et al. found that inhibiting the entry of Ly6C^high^, but not Ly6C^low^ monocytes, into the atherosclerotic plaque during normocholesterolemia abrogates atherosclerosis regression (161). Analogous to their phenotype in the steady-state, Ly6C^high^ monocytes might not necessarily differentiate into macrophages, but instead participate in tissue repair with their monocyte-specific functions. In a model of sterile liver injury, as well as during the resolution phase after myocardial infarction, recruited classical Ly6C^high^ monocytes performed a phenotypic switch to non-classical Ly6C^low^ monocytes, which was crucial for optimal tissue repair (99, 157). The precise functions of circulating Ly6C^low^ monocytes during atherosclerosis regression have not yet been clarified. Given their role in the integrity of the endothelium, it is conceivable that intravascular Ly6C^low^ monocytes participate in the reorganisation of the endothelial layer during the plaque size reduction. It will be interesting to see first results of studies focussing on the role Ly6C^low^ monocytes during atherosclerosis regression, for instance in a mouse model with a Ly6C^low^ monocyte-specific deficiency (162).

Akin to the inflammation-poised phenotype of monocytes circulating during hypercholesterolemia and atherosclerosis development described above, resolution-dedicated monocyte subsets have been found to be present in the inflammatory resolution of sepsis and colitis (163). However, as to whether the reparatory Ym1 (chitinase-like protein 3)^+^Ly6C^high^ monocyte subset described is also present during atherosclerosis resolution has not been investigated. Nevertheless, Ly6C^high^ monocytes have been found to exhibit an altered surface expression of various proteins during the regression of atherosclerosis (146). This underlines the importance of the quality over the quantity of the monocyte response and offers an explanation as to why atherosclerosis regression continues undisturbed in studies with suppressed monocyte recruitment.

Emerging scRNA-seq studies of atherosclerosis regression have been providing us with an insight regarding the heterogeneity of the remaining and recruited macrophages in regressing plaques (48, 149). Interestingly, the same, previously mentioned, five main macrophage clusters present during atherosclerosis have also been observed in regressing plaques (48, 70, 149). This might be less surprising for the subsets of cavity-like and TREM2^+^ macrophages. As mentioned above, cavity macrophages have been found to be essential mediators of tissue repair (127–129). The scRNA-seq studies of atherosclerosis regression, however, are unable to inform us about the location of the analysed macrophages, and thus it is unclear as to whether these cavity macrophages have invaded the intima, or if they participate in the resolution of the intimal inflammation However, TREM2^+^ macrophages are known to be equipped for lipid handling, and the accumulation of extracellular lipids is part of the tissue repair when dead and apoptotic cells need to be cleared by efferocytosis, a process that is increased in regressing plaques (149).

Although these studies identified the same major macrophage clusters in regressing plaques as in atherosclerosis development, there were subtle differences in expression levels representing a spectrum of activation states (48, 149). The subset of inflammatory macrophages, for instance, showed decreased expression levels of *Il1β* and *Nlrp3*, compared to atherosclerotic macrophages before the induction of atherosclerosis regression (149). Interestingly, the described interferon-inducible macrophages had increased transcription levels of *signal transducer and activator of transcription 6* (*Stat6)*, which is known to induce type 2 or reparatory immune responses (149). Notably, when atherosclerotic aortic segments were transplanted in normocholesterolemic Stat6-deficient mice, atherosclerosis regression was abrogated, which was associated with a pro-inflammatory phenotype of plaque macrophages (161). A question that has not been finally resolved is if the already present plaque macrophages can be repolarized by the regressing conditions to adjust their functional program towards a reparatory phenotype or if an influx of *de novo* reparatory macrophages is required. The study by Rahman et al. found that Ly6C^high^ monocyte influx is an absolute requirement for plaque regression and differentiation of reparatory macrophages (161). This is in line with evidence that inflammatory macrophages cannot be repolarized to reparatory macrophages (164) undefined. In other reports inflammatory macrophages could be repolarized to a reparatory phenotype, although only a limited number of phenotypic markers were assessed (165, 166). Furthermore, an elegant *in vivo* tracking approach found a phenotypic adjustment of individual macrophages from inflammatory inducible nitric oxide synthase-expressing to arginase-expressing macrophages in a model of chronic central nervous inflammation (167). If and to what extent a local phenotypic switch of macrophages occurs in the regressing atherosclerotic plaque remains elusive and warrants further studies.

Interestingly, when Lin et al. broke down the transcriptional differences in macrophages during progression and regression in more detail, they identified one substantial macrophage subset and 42 distinctly regulated genes that were predominantly present during regression. The macrophage subset was characterised by high expression of *Stab1*, which is important for efferocytosis. In addition to *Stab1*, *Gas6* represents another upregulated molecule important for efferocytosis (48). Intriguingly, we have previously found that adventitial EMP-derived macrophages are characterised by high expression levels of both Stab1 and Gas6 (37). The presence of a regression-specific macrophage subset expressing a similar signature might indicate a role for EMP-derived macrophages in the tissue repair during atherosclerosis regression, and even hint towards the migration of these prenatally seeded adventitial macrophages into the intima. Relatedly, EMP-derived macrophages are known to be important regulators of tissue repair in the heart (168, 169). So far, it was assumed that adventitial macrophages do not cross the media and immigrate into the intima, but future studies will have to re-evaluate the fate of adventitial EMP-derived macrophages during atherosclerotic disease.

## Human Translatability

The wide array of available methods, including genetic fate-mapping models, intravital imaging or tracking of adoptively transferred cells, makes the mouse an ideal model system for studying the trafficking behaviour and dynamics of monocytes and macrophages. Although these models allow us to study the trafficking of mononuclear phagocytes in the mouse vasculature, ultimately the goal is to advance our understanding of these features in the human-being. Since similar scientific manipulations are unfeasible in the human, descriptive studies are used to determine the translatability of results in the mouse to the human situation.

In mice YS EMP-derived macrophages seed the aorta during early embryonic development. Haematopoiesis is a conserved process between men and mice, with an initial haematopoietic wave originating in the extra-embryonic YS, followed by a transition to intra-embryonic definitive haematopoiesis (170, 171). We and others have previously identified primitive macrophages in the human YS that show a phenotype similar to mouse EMP-derived macrophages in the mouse (27, 172, 173). A recent study employing scRNA-seq on human embryonic tissue at different time points of organogenesis found tissue-resident macrophages originating from the YS as well as the fetal liver (174), thus providing evidence for an initial seeding of vascular macrophage during early human embryogenesis and corroborating rodent studies. A second wave of monocyte-derived macrophage presumably follows the initial seeding with YS-derived macrophages in the human. Direct evidence for this in the arterial wall is lacking but studies in other human tissues were able to translate the results of mouse studies to humans. Langerhans cells in the skin have been shown to be YS-derived resident epidermal macrophages seeding the tissue in the first wave, whereas dermal macrophages are of monocytic origin in mice (31, 175). In humans with an inherited severe monocytopenia, a dramatically reduced frequency of CD14^+^ dermal macrophages but sustained numbers of Langerhans cells could be observed (176, 177). The skin is an easily accessible organ with different macrophage ontogenies that enables investigation of macrophage trafficking in humans. Other future options might include the study of conserved epigenetic marks between the murine and human system also in the cardiovascular system and especially the arterial wall.

Like in the mouse, macrophages are a major subset or even the dominant immune subset in the non-atherosclerotic arterial wall of humans, although the human arteries also contain significant numbers of T lymphocytes (16, 37, 42, 43, 178–180). Arterial phagocytes can be found in the intima, directly beneath the endothelial layer, mirroring their function as immune sentinels, as well as in the adventitia (2, 180–185). Arterial resident macrophages are more prevalent in the adventitial layer than in the intima, although the difference is less pronounced compared to the mouse (186, 187). As would be expected for immune sentinels, intima-resident macrophages can be found more frequently at atheroprone sites, which show non-laminar and low shear stress blood flow (2, 180, 183). Studies in other human organs have provided evidence that tissue-resident macrophages self-sustain mainly through local proliferation without monocyte input, although there might be differences depending on the macrophage subset. In studies of sex-mismatched hand allografts, YS-derived Langerhans cells were not replaced by recipient cells but remained of donor origin up to 10 years post-transplantation (188, 189). This is in line with results of sex-mismatched heart transplants, where only 31% of presumably BM-derived CCR2^+^ macrophages were of recipient origin compared to less than 1% CCR2^-^, potentially YS-derived, resident macrophages, after a mean period of 8.8 years post-transplantation (190). The arterial wall of the vessels in the transplanted organs has not been examined separately, but a recent scRNA-seq study of human healthy arterial tissue identified a proliferative macrophage subset (178), hinting towards a self-sustaining arterial resident macrophage population.

Although the human intima harbours subendothelial macrophages and CD11c^+^ phagocytes that mirror the recently identified aortic intima-resident macrophage of the mouse (16, 181, 191), there are important differences in the intimal composition between mouse models and humans that need to be considered. The human intima is thickened and comprises abundant VSMCs and extracellular matrix at sites prone to atherosclerotic development. Additionally, VSMCs are very plastic cells and in addition to being producers of extracellular matrix components can be phagocytic and develop into foam cells. The discrimination of VSMC and macrophage foam cells is complicated by the fact that, VSMCs can express macrophage markers like CD68, whereas macrophages have also been found to express VSMC lineage markers (51, 192–195). Lineage-tracing studies in mice have shown a varying degree of foam cells originating from VSMCs, ranging from 16% to 70% (53, 195–197). In humans, it has been estimated through the analysis of histone marks that 18% of CD68^+^ plaque cells originate from the VSMC lineage (51). Future studies will have to determine to what extent VSMCs and macrophages contribute to the foam cell pool at different phases of the atherosclerotic process.

The inflammatory reaction following the influx, retention and modification leads to a continuous recruitment of human monocytes into the growing atherosclerotic lesion (198, 199). Evidence for an important role of monocyte recruitment in the development of human atherosclerosis derives from studies showing an association of monocyte counts with atherosclerotic plaque development during several years of follow-up (200–202). Human monocytes can be distinguished into three different subsets: (I) classical CD14^+^CD16^-^, analogous to the Ly6C^high^ mouse population, (II) non-classical CD14^dim^CD16^+^, aligning with the murine Ly6C^low^ subset, and (III) intermediate CD14^+^CD16^+^ monocytes (203). Emerging results from multiparametric analyses identified further subsets and it will be interesting to determine their functional relevance in atherosclerosis (204–206). The non-classical CD14^dim^CD16^+^ monocytes fulfil similar endothelial surveillance functions as in the mouse, whereas the role of intermediate monocytes is not yet clear in the atherosclerotic progress (55–57, 203). The classical CD14^+^CD16^-^ are thought to mainly enter the growing atherosclerotic lesion (207), as this subsets preferentially migrates into tissues and differentiates to macrophages (208–211). Consequently, it has been shown that higher numbers of circulating CD14^+^CD16^-^ monocytes predict cardiovascular events (212, 213). Interestingly though, classical CD14^+^CD16^-^ monocytes do not associate with a more high-risk plaque phenotype in patients with advanced atherosclerosis (214). This might be owed to a more important role of local macrophage proliferation than monocyte recruitment in advanced atherosclerosis, similar to what has been observed in mice. In line with this, advanced atherosclerotic plaques contain a significant fraction of proliferating macrophages (71, 215–219). Another striking similarity between the human and mouse plaque macrophages relates to their phenotype. An integrated analysis of scRNA-seq subsets of the mouse and human revealed a conserved phenotype between the two species, with detection of (I) inflammatory, (II) foamy TREM2^+^, (III) resident-like and (IV) interferon-inducible macrophages (71).

In summary, there are important differences between human and mouse atherosclerosis, as exemplified by the presence of a thickened VSMC-rich intima in the human arterial wall. Nonetheless, studies in rodent models have been instructive in examining basic principles of the trafficking of mononuclear phagocytes and will continue to provide valuable insight. Novel techniques, such as spatial transcriptomics (220), hold a great promise in translating murine results to the human situation.

## Conclusion and Outstanding Questions

Monocytes and macrophages are key effector cells during all phases of atherosclerotic disease. Their trafficking in and out of the arterial wall directly influences the disease process. Although we have gained substantial insight into these processes during atherosclerosis development, there are still major gaps in our knowledge. For instance, it is currently unknown if invading monocytes persist in a non-differentiated state within the plaque or if their only fate is the differentiation to plaque macrophages. Answering this question is complicated by the phenotypic similarities of monocytes and macrophages. The combination of newly developed fate-mapping models with novel methodologies, like spatial transcriptomics, display a promising avenue for future investigations of cellular fates within the plaque. Along these lines, the recently identified subset of intima-resident macrophages illustrates the potential of such methodologies to deciphering macrophage dynamics within the arterial wall by using novel methodologies.

Nonetheless it is unclear if intima-resident macrophages vanish entirely upon onset of hypercholesterolemia or can rebound once cholesterol levels are normalised. Another remaining question relates to the dynamics of replacing intima-resident macrophages by recruited macrophages. Does the resident subset die, emigrate or just stop its proliferation?

Another major remaining question is the role of adventitia-resident macrophages in atherosclerosis. During atherosclerosis development, perinatally seeded intima-resident macrophages are quickly replaced by recruited inflammatory macrophages. The replacing cells are presumably transient macrophages, which do not engraft after inflammation resolution. As mentioned earlier, it is currently unclear whether a small subset of intima-resident prevails during atherosclerosis progression, and whether these cells are capable of rebounding following the cessation of hypercholesterolemia. Consequently, adventitial macrophages might be the only long-term resident macrophages in the aorta during atherosclerosis development. In contrast to the recruited inflammatory macrophages in the intima, adventitial macrophages do not show increased proliferation during atherosclerosis development, resulting in largely stable macrophage numbers despite the continuous inflammation in the local environment (49). This, and the low migratory capacity of plaque macrophages could argue for a limited role of adventitia-resident macrophages in the atherosclerotic process. On the other hand, regressing atherosclerotic plaques contain a subset of macrophages possessing a transcriptional signature that is reminiscent of homeostatic and pro-resolving EMP-derived adventitial macrophages. Future studies will have to evaluate the role of adventitial macrophages in atherosclerosis and investigate if these cells are capable of migration into the intima. Given their pro-resolving phenotype, adventitial EMP-derived macrophage and their migration into the atherosclerosis-affected intima also display a potential therapeutic target.

In general, we are lacking studies that quantitatively examine the recruitment and different fates of monocytes and macrophages during the different phases of atherosclerosis progression, and in particular during atherosclerosis regression. Novel fate mapping and conditional gene deletion models, such as the Ms4a3^cre^ (30), CCR2^cre^ (221–223) and Rank^cre^ (45) mice, together with high-dimensional analysis approaches will aid in deepening our understanding of these processes.

In this Review, we have mainly focused on results from mouse models but summarized evidence for similarities as well as differences between the rodent and human arterial wall. Many aspects pertaining the trafficking of monocytes and macrophages are difficult to corroborate in humans, given the unfeasibility of fate-mapping techniques. Nonetheless, the development of novel methods, including scRNA-seq and spatial omics-technologies will continue to expand the possibilities of analysing monocyte and macrophage dynamics in humans. Although several findings in the mouse can be translated to the human, there are differences in the pathological mechanisms, which call for an increased effort in performing human studies.

## Author Contributions

LT and CS conceived the idea and article structure. FP and LT wrote and edited the manuscript. FP created the illustrations and CS revised the manuscript and provided oversight. All authors have made a substantial, direct and intellectual contribution to the article and approved the submitted version.

## Funding

LT is supported by a Walter Benjamin fellowship of the German Research Foundation (DFG). This study was supported by the DFG, SFB 1123 project A07 to CS.

## Conflict of Interest

The authors declare that the research was conducted in the absence of any commercial or financial relationships that could be construed as a potential conflict of interest.

## Publisher’s Note

All claims expressed in this article are solely those of the authors and do not necessarily represent those of their affiliated organizations, or those of the publisher, the editors and the reviewers. Any product that may be evaluated in this article, or claim that may be made by its manufacturer, is not guaranteed or endorsed by the publisher.
